# Synaptic plasticity and spatial working memory are impaired in the CD mouse model of Williams-Beuren syndrome

**DOI:** 10.1186/s13041-016-0258-7

**Published:** 2016-08-02

**Authors:** Cristina Borralleras, Susana Mato, Thierry Amédée, Carlos Matute, Christophe Mulle, Luis A. Pérez-Jurado, Victoria Campuzano

**Affiliations:** 1 Departament de Ciències Experimentals i de la Salut, Universitat Pompeu Fabra, Barcelona, Spain; 2 Neurosciences Program, Institut Hospital del Mar d’Investigacions Mèdiques (IMIM), Barcelona, Spain; 3 Centro de Investigación Biomédica en Red de Enfermedades Raras (CIBERER), ISCIII, Madrid, Spain; 4 Department of Neuroscience, Neurotek-University of the Basque Country, Leioa, Spain; 5 Achucarro Basque Center for Neuroscience, Zamudio, Spain; 6 Centro de Investigación Biomédica en Red de Enfermedades Neurodegenerativas (CIBERNED), Madrid, Spain; 7 Interdisciplinary Institute for Neuroscience, CNRS UMR 5297 - University of Bordeaux, F-33000 Bordeaux, France

**Keywords:** Williams-Beuren syndrome, Mouse model, Memory, Synaptic plasticity, LTP, Hippocampus

## Abstract

Mice heterozygous for a complete deletion (CD) equivalent to the most common deletion found in individuals with Williams-Beuren syndrome (WBS) recapitulate relevant features of the neurocognitive phenotype, such as hypersociability, along with some neuroanatomical alterations in specific brain areas. However, the pathophysiological mechanisms underlying these phenotypes still remain largely unknown. We have studied the synaptic function and cognition in CD mice using hippocampal slices and a behavioral test sensitive to hippocampal function. We have found that long-term potentiation (LTP) elicited by theta burst stimulation (TBS) was significantly impaired in hippocampal field CA1 of CD animals. This deficit might be associated with the observed alterations in spatial working memory. However, we did not detect changes in presynaptic function, LTP induction mechanisms or AMPA and NMDA receptor function. Reduced levels of Brain-derived neurotrophic factor (BDNF) were present in the CA1-CA3 hippocampal region of CD mice, which could account for LTP deficits in these mice. Taken together, these results suggest a defect of CA1 synapses in CD mice to sustain synaptic strength after stimulation. These data represent the first description of synaptic functional deficits in CD mice and further highlights the utility of the CD model to study the mechanisms underlying the WBS neurocognitive profile.

**Electronic supplementary material:**

The online version of this article (doi:10.1186/s13041-016-0258-7) contains supplementary material, which is available to authorized users.

## Introduction

Williams-Beuren syndrome (WBS, OMIM 194050) is a rare neurodevelopmental disorder caused by a microdeletion of 26-28 genes on chromosome 7q11.23 with an estimated prevalence of 1 in 7500 [1]. Individuals with WBS present mild to moderate intellectual disability with an average intelligence quotient (IQ) of 55 (ranging from 40 to 100) [2–4]. The syndrome is characterized by an unusual cognitive profile that includes relatively preserved expressive language and facial processing abilities but dramatic deficits in spatial cognition [4–6]. Processing of spatial navigational information and verbal long-term memory, domains highly dependent on hippocampal function, are also severely affected in WBS [7–9]. In addition to this clinical evidence of hippocampal dysfunction, structural and functional abnormalities have also been reported in WBS [10]. Although the global volume of the hippocampus is preserved, WBS individuals present a shape abnormality in the midsection of the hippocampus and functional studies revealed an overall depression of hippocampal energy metabolism and synaptic activity [10].

The complete deletion (CD) mouse model was generated in order to mimic the most common and recurrent deletion found in WBS patients, encompassing all single copy genes of the syntenic interval from *Gtf2i* to *Fkbp6* [11]. CD mice presented with many features reminiscent of WBS such as growth deficiency, craniofacial and cardiovascular abnormalities, and several behavioral alterations including hypersociability. In addition, CD mice presented an increase proportion of immature neurons in the dentate gyrus together with shorter dendrites and decreased spine density in CA1 pyramidal neurons of the hippocampus [11].

Among the genes deleted in WBS, *GTF2I, CLIP2 LIMK1 and STX1A* are good candidates for some of the neurobehavioral features of WBS [12–17]. Knockout mouse models of *Clip2*, *Limk1* and *Stx1a* presented impairment in hippocampal long-term potentiation (LTP) that correlated with behavioral alterations, specifically hippocampal-dependent memory deficits [14, 16, 18, 19]. A *Gtf2i* mouse model presented several behavioral alterations such as hypersociability and anxiety-related behavior together with a reduction in spine density in hippocampal pyramidal neurons [20, 21].

Although single-gene knockout mouse models have helped to elucidate the contribution of individual genes to the complex phenotype associated with WBS, the CD mouse model might be more closely related to the human phenotype. Therefore, we focused our investigations on hippocampal-dependent synaptic plasticity and memory in the CD model. We have studied cognitive function with a test sensitive to hippocampal function and analyzed the synaptic function on hippocampal slices. CD mice showed deficits in the spontaneous alternation test, indicating impairment in spatial working memory. In addition, electrophysiological experiments showed a significant reduction in the LTP in CA1 hippocampus synapses of CD mice, which could be associated with the reduced levels of BDNF observed in these mice. Taken together, these findings further reinforce the notion that functions controlled by the hippocampus are impaired in CD mice and underlie some of the behavioral deficits present in this animal model of WBS.

## Results

### Spatial working memory is impaired in CD mice

**Fig. 1:**
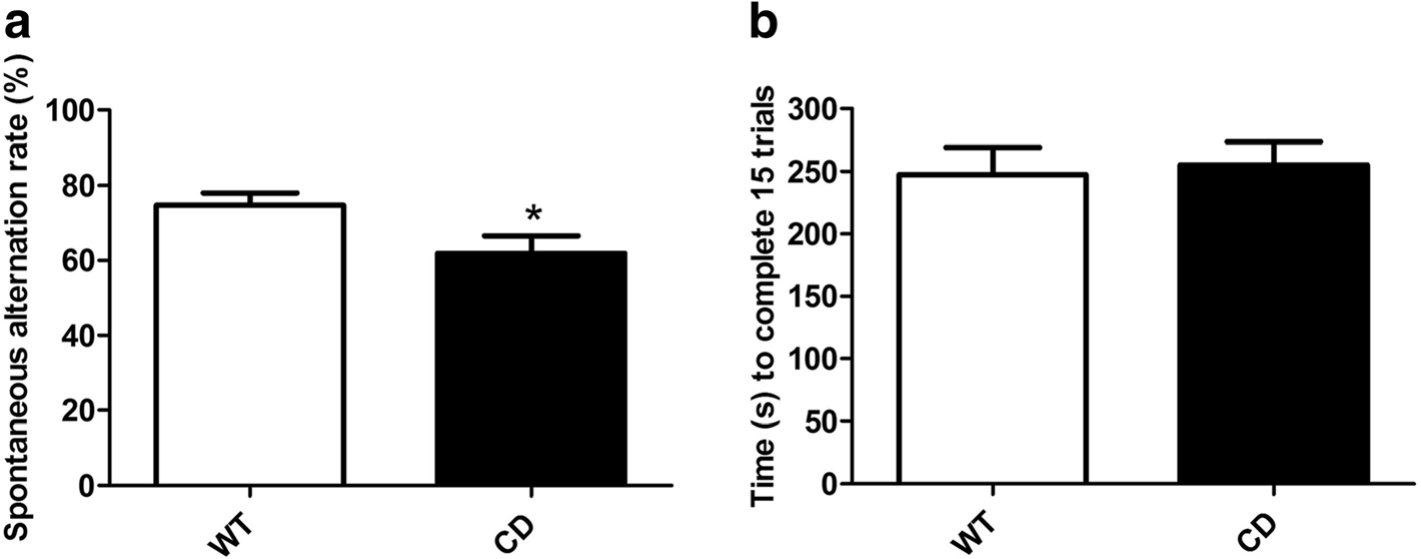
Impaired spatial working memory in CD mice. **a** The percentage of alternation in the spontaneous alternation test was significantly reduced in CD mice compared to WT mice (*p* = 0.0282, unpaired *t* test). **b** No differences were found (*p* = 0.789, unpaired *t* test) in the total time to complete 15 choices/trials in the spontaneous alternation test. WT: *n* = 13; CD: *n* = 9. Data are presented as the mean ± SEM. *p* values are shown with asterisks indicating values that are significantly different. *p < 0.05

WBS individuals present working memory impairment, especially in spatial working memory [7, 22]. We tested whether this phenotype is also present in CD mutants using the spontaneous alternation test. This behavioral test has been widely used to study spatial working memory and it has been shown to be very sensitive to hippocampus dysfunction, although other brain areas are also involved in the task. It is based on the natural tendency of mice to explore novel environment [23–25]. Therefore, mice typically prefer to investigate a new arm rather than returning to the arm they have just visited, showing an alternation behavior [25]. Usually, the alternation rate of a wild-type (WT) mouse is around 75 %. CD mice displayed spontaneous alternation of 61.90 % ± 3.20 of choices, which was significantly lower than 74.73 % ± 4.61 of alternation observed in the WT mice (*p* = 0.0282, unpaired *t* test) (Fig. 1a). To differentiate whether differences in alternation could be related to locomotor or exploratory activity, time to complete the 15 choices was also recorded. There were no differences in the total time to complete the test between WT (247.47 ± 21.56 s) and CD mice (255.29 ± 18.39 s) (*p* = 0.798, unpaired *t* test) (Fig. 1b).

### CD mice exhibit deficits in LTP

**Fig. 2:**
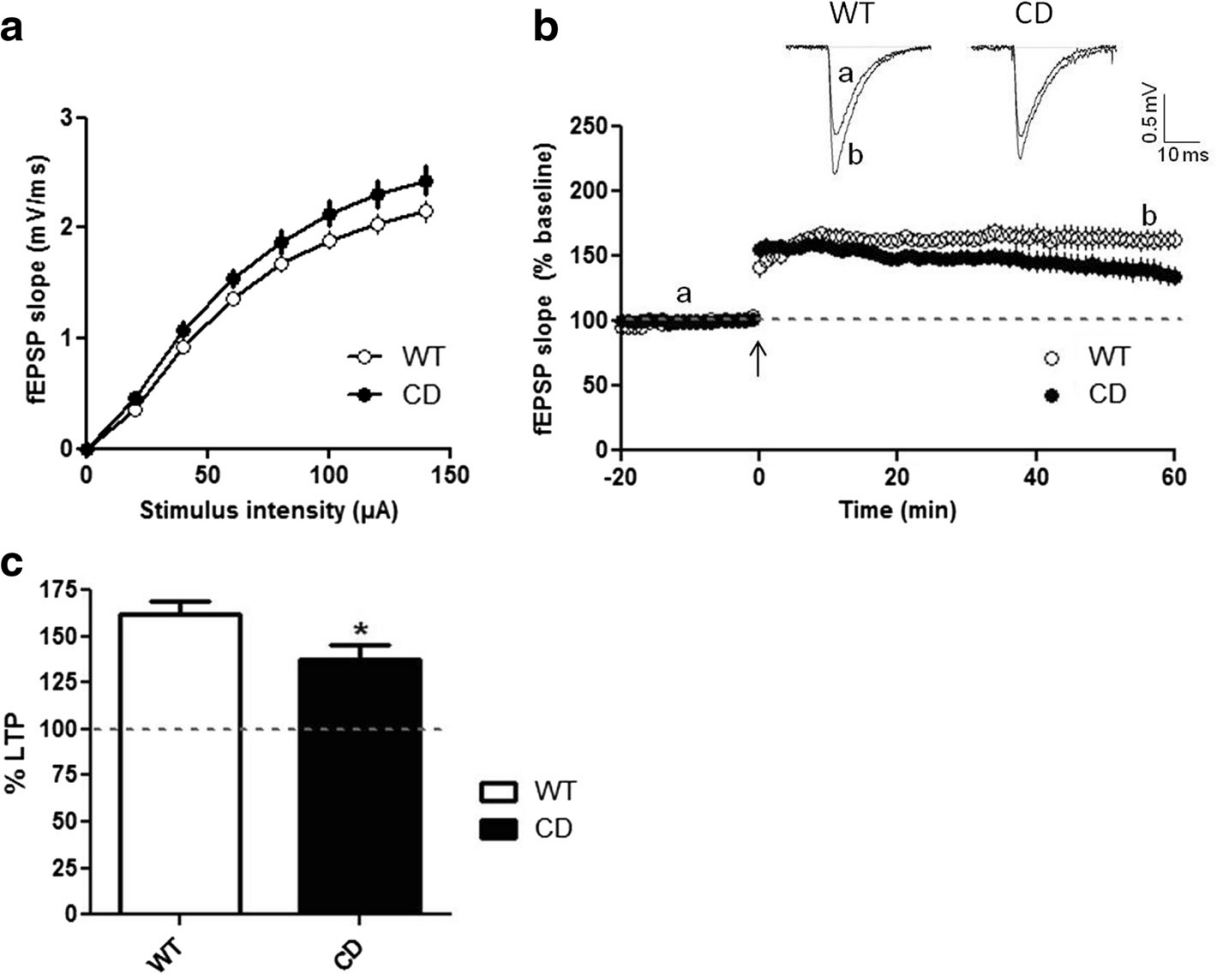
Hippocampal LTP is impaired in CD mice. **a** Input-output curves at the CA3-CA1 hippocampal synapse. The stimulating electrode was placed in the Schaffer collateral fibers and the recording electrode in the stratum radiatum of the CA1 region. Two-way repeated measures ANOVA revealed no differences between genotypes (*p* = 0.063). WT: *n* = 20, CD: *n* = 21. **b** Time course of normalized fEPSPs showing the effect of TBS (arrow, time 0) in WT and CD mice. Data are expressed as the percentage of mean fEPSP slopes recorded during the baseline period. Insert shows overlaid representative fEPSP traces taken at the indicated time points. WT: *n* = 12, CD: *n* = 13. **c** The extent of synaptic potentiation 55–60 min after TBS was significantly reduced in CD mice (*p* = 0.035, unpaired *t* test) compared to WT mice. WT: *n* = 12, CD: *n* = 13. Data are presented as the mean ± SEM. *p* values are shown with asterisks indicating values that are significantly different. *p < 0.05

To investigate the electrophysiological consequences of the CD mutation we performed recordings in the CA1 region of the hippocampus in comparison to WT animals. Basal synaptic transmission was studied over a range of stimulus intensities and was analyzed in terms of field excitatory postsynaptic potentials (fEPSP) slope versus stimulus intensity to test for general deficits in synaptic connectivity. Input-output curves for fEPSP elicited by stimulation of Schaffer commissural projections were not detectably different between CD and WT mice (*p* = 0.063, two-way repeated measures ANOVA) (Fig. 2a). We next focused on analyzing LTP as this form of activity-dependent synaptic plasticity is widely acknowledged as a physiological substrate of information storage in the hippocampus. To study LTP we investigated the effects of theta burst stimulation (TBS) in the CA1 region of hippocampal slices from control and mutant animals. As shown in Fig. 2b, 5 trains of 10 theta bursts induced a persistent enhancement of synaptic transmission that remained stable during the entire period of recording in WT mice (61.92 % ± 6.96 at 55–60 min after TBS). In contrast, synaptic potentiation following TBS decayed slowly in CD mice (Fig. 2b) and the magnitude of LTP at the end of the recording was significantly reduced from that measured in WT mice (37.38 % ± 8.41; *p* = 0.035, unpaired *t* test) (Fig. 2c).

### Unaltered presynaptic function in CD mice

**Fig. 3:**
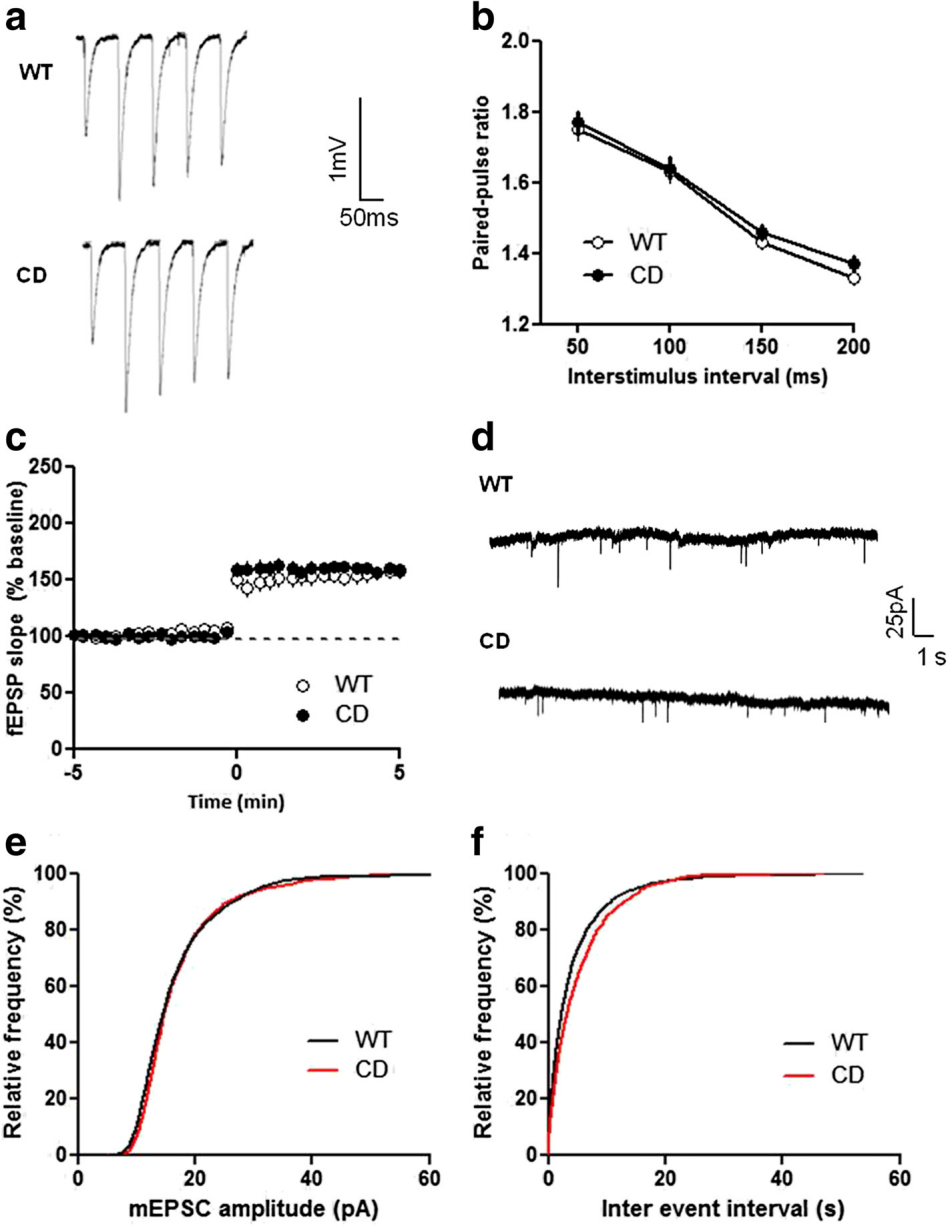
Presynaptic function and AMPA receptor-mediated synaptic transmission are not altered in CD mice. **a** Representative traces of PPF at different interstimulus intervals. **b** The ratio of PPF was obtained by dividing the amplitude of the second fEPSP by the amplitude of the first fEPSP. Two-way repeated measures ANOVA revealed no differences between genotypes (*p* = 0.37). WT: *n* = 19, CD: *n* = 20. **c** Normal PTP in CD mice. TBS was given at t = 0 and the fEPSP were recorded immediately after the tetanus. WT: *n* = 12, CD: *n* = 14. **d** Representative traces of miniature mEPSCs from WT and CD neurons. **e** Cumulative probability analysis of the distribution of inter-event intervals between mEPSCs showed no differences between WT and CD mice (*p* = 0.341, Kolmogrov-Smirnov test). **f** Cumulative probability analysis of mEPSCs amplitude showed no differences between WT and CD mice (*p* = 0.745, Kolmogrov-Smirnov test). WT: *n* = 22, CD: *n* = 12. Data are presented as the mean ± SEM

We next attempted to identify which of the events mediating LTP production were altered in CD mice. Changes in the presynaptic efficacy of excitatory transmission may cause alterations in short- and long-term plasticity and have been proposed to contribute to LTP abnormalities [26, 27]. To examine if CD mice exhibited alterations in the properties of neurotransmitter release we first looked for possible changes in paired-pulse facilitation (PPF), a short lasting enhancement in synaptic strength mediated by presynaptic calcium-dependent mechanisms so that the response to a second stimulation is potentiated when delivered within 200 ms of the first stimulus [28]. Schaffer collateral-CA1 synapses were briefly stimulated twice with varied inter-stimulus intervals and fEPSPs were recorded. As shown in Fig. 3a and b, the degree of PPF at interpulse intervals of 50, 100, 150 and 200 ms was indistinguishable between WT and CD mutant slices (*p* = 0.37, two-way repeated measures ANOVA). Consistently, whole cell recordings of PPF using an interstimulus interval of 50 ms revealed no differences between WT and CD mice (Additional file 1: Figure S1). Analysis of synaptic responses immediately after TBS demonstrated that post-tetanic potentiation (PTP), a form of short-term plasticity mediated by changes in presynaptic calcium entry or in the release machinery [29], was also identical between the two groups of mice (*p* = 0.24, two-way repeated measures ANOVA for minutes 0–2 after TBS) (Fig. 3c).

LIMK1 inactivation leads to alterations in neurotransmitter release evidenced as changes in the dynamics of miniature excitatory postsynaptic currents (mEPSCs) despite the absence of major modifications in the degree of PPF or PTP [18]. Thus, we next analyzed the frequency and amplitude of mEPSCs from CA1 pyramidal neurons in CD mice (Fig. 3d). We found that the rate of mEPSCs was similar between genotypes (WT:0.22 ± 0.02 Hz, CD:0.20 ± 0.03 Hz), further supporting the absence of major modifications in the probability of vesicle fusion or in the number of synapses on CA1 neurons (Fig. 3e). The amplitude of mEPSCs was also identical between the two groups of mice (WT:14.82 ± 0.56 pA, CD: 14.22 ± 0.45 pA), suggesting preserved AMPA receptor function in the CD mutants (Fig. 3f).

### LTP induction is preserved in CD mice

**Fig. 4:**
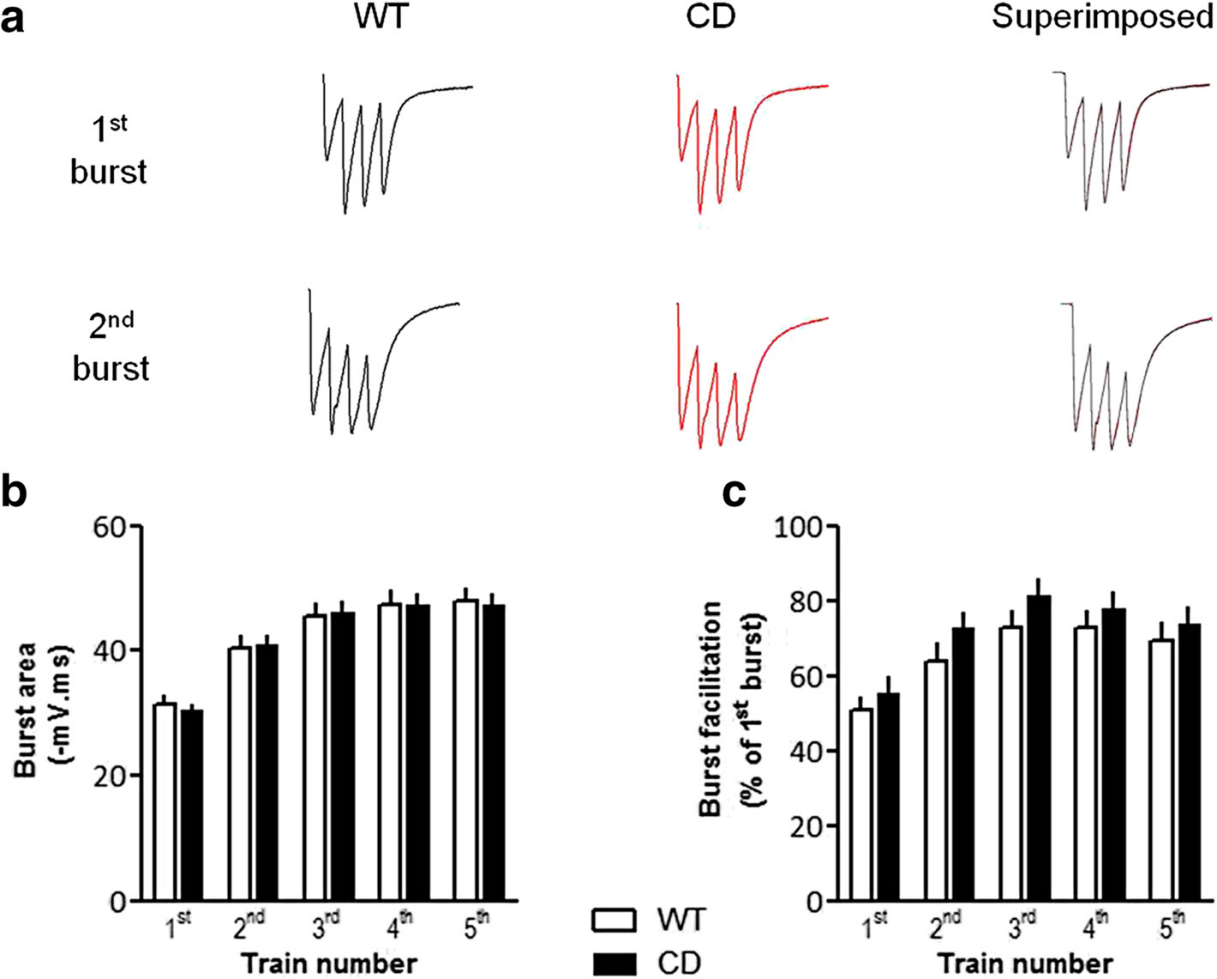
CD mice exhibit normal LTP induction mechanisms. **a** Waveforms of the postsynaptic responses generated by the 1^st^ and 2^nd^ theta burst in the first train of the TBS protocol. Note that the second burst response in each case is larger than the first and does not return as quickly to baseline. Comparison of the averaged composite fEPSPs recorded from WT and CD slices indicates that the mutation does not affect the waveform of the response or its transformation within the train. **b** Mean sizes of initial burst responses in each train was similar in WT and CD mice (*p* = 0.96, two-way repeated measures ANOVA). **c** Facilitation of burst responses was calculated by expressing the area of the composite fEPSP corresponding to the 2nd theta burst within each train as a fraction of the 1st burst response. Two-way repeated measures ANOVA revealed no differences between genotypes (*p* = 0.30). WT: *n* = 12, CD: *n* = 16. Data are presented as the mean ± SEM

We next attempted to identify whether the mechanisms involved in LTP induction were negatively affected in CD mice. Earlier work has shown that bursts repeated at the frequency of the endogenous theta rhythm induce maximal LTP. This frequency disables a feed-forward inhibitory postsynaptic potential (IPSP) activated by the first burst and truncates the EPSPs evoked by that burst or by subsequent bursts evoked within 100–150 ms. Once being activated by a first burst, feed-forward IPSPs become suppressed due to the activation of presynaptic GABA_B_ auto-receptors that results in diminished GABA release [30, 31]. Because of this disinhibition, a second burst at 200 ms after the first burst evokes maximal postsynaptic depolarization needed to unblock postsynaptic NMDA receptors and allow a calcium influx into dendritic spines that serves as the proximal trigger for LTP [32]. As a first approach to evaluate possible defects in LTP induction mechanisms we analyzed theta bursts responses in CD mutants. There were no evident between-group differences in the waveforms of the composite postsynaptic responses generated by theta burst as shown from the averaged traces in Fig. 4a. The mean sizes of the initial burst responses were comparable for WT and CD mice (*p* = 0.96, two-way repeated measures ANOVA) as was the degree to which the second theta burst is facilitated in each train (*p* = 0.30, two-way repeated measures ANOVA) (Fig. 4b and c). These data suggest that GABA_A_ receptor mediated IPSPs as well as the after hyperpolarizations that follow cell spiking are not significantly different between genotypes.

### Normal AMPA/NMDA ratio of excitatory postsynaptic currents in CD mice

**Fig. 5:**
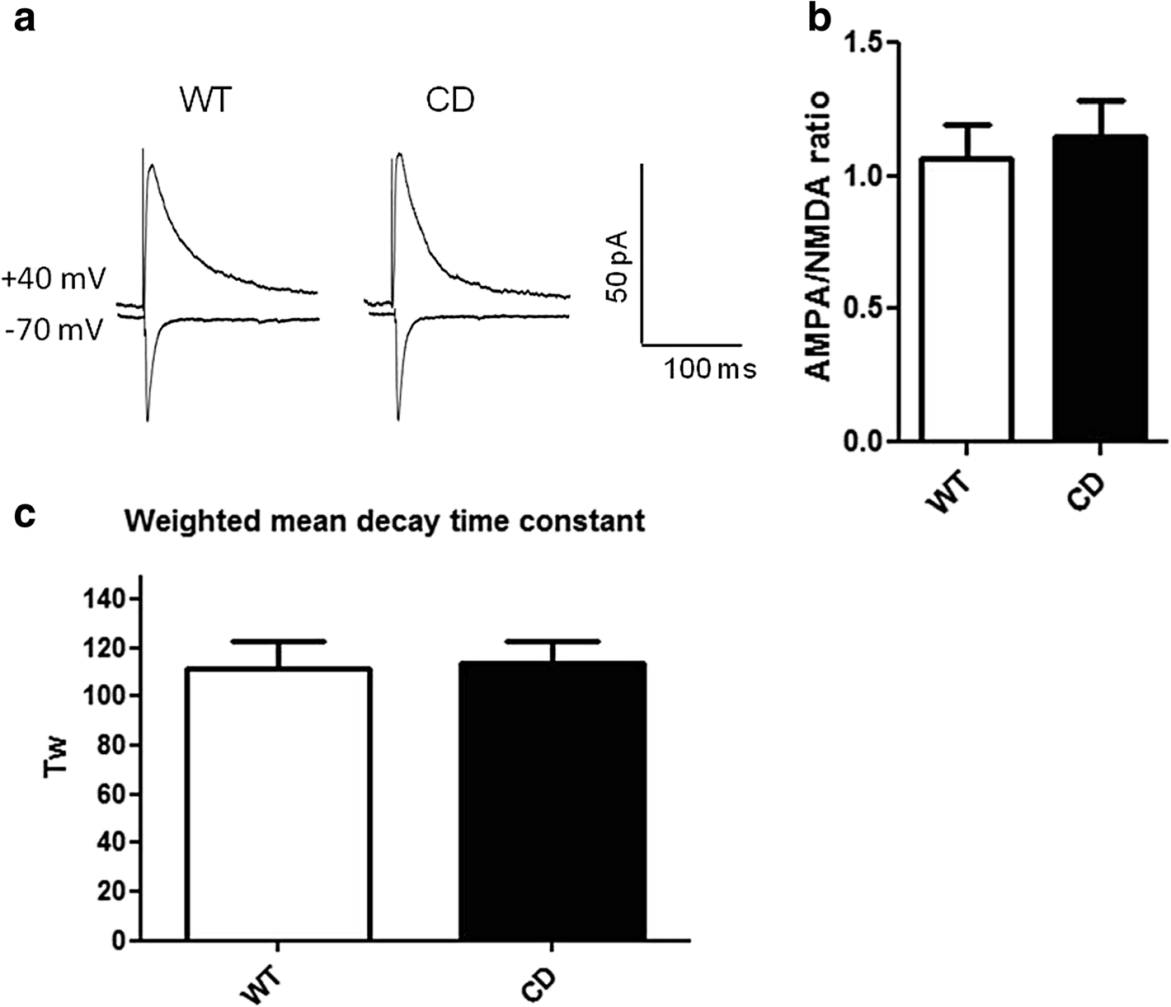
AMPA/NMDA ratio at Schaffer collateral – CA1 synapse. **a** Representative WT and CD traces of AMPA and NMDA receptor-mediated response recorded in the same cell at -70 and +40 mV, respectively. **b** The AMPA receptor current amplitude was calculated at the peak at a holding potential of −70 mV and the NMDA receptor current amplitude was measured 40 ms after stimulation artifact at a holding potential of +40 mV. The AMPA/NMDA ratio in CD neurons was not significantly different (*p* = 0.643, unpaired *t* test) from WT neurons. WT: *n* = 13, CD: *n* = 14. **c** Weighted mean decay time constant (Tw) of EPSCs recorded at +40 mV was not different (*p* = 0.455, unpaired *t* test) between WT and CD neurons. WT: *n* = 13, CD: *n* = 12. Data are presented as the mean ± SEM

A differential synaptic expression or subunit composition of NMDA receptors can set the threshold capacity to express NMDA receptor-dependent LTP in rodent models of mental disability and autism. This idea is supported by several studies showing defects in LTP that correlate with decreased NMDA receptor protein levels or changes in the AMPA/NMDA receptor ratio in fragile X mental retardation-1 and neuroligin-1 knockout mice [33–35]. Thus we next quantified and compared the ratio of evoked synaptic AMPA and NMDA receptor currents in CA1 pyramidal neurons of WT and CD mutants. We could not appreciate any difference in AMPA/NMDA ratio between genotypes (Fig. 5a and b), nor significant alterations in the decay time of NMDA receptor mediated EPSCs recorded at +40 mV between WT and CD mice (Fig. 5c).

### Reduced BDNF levels in hippocampal neurons of CD mice

**Fig. 6:**
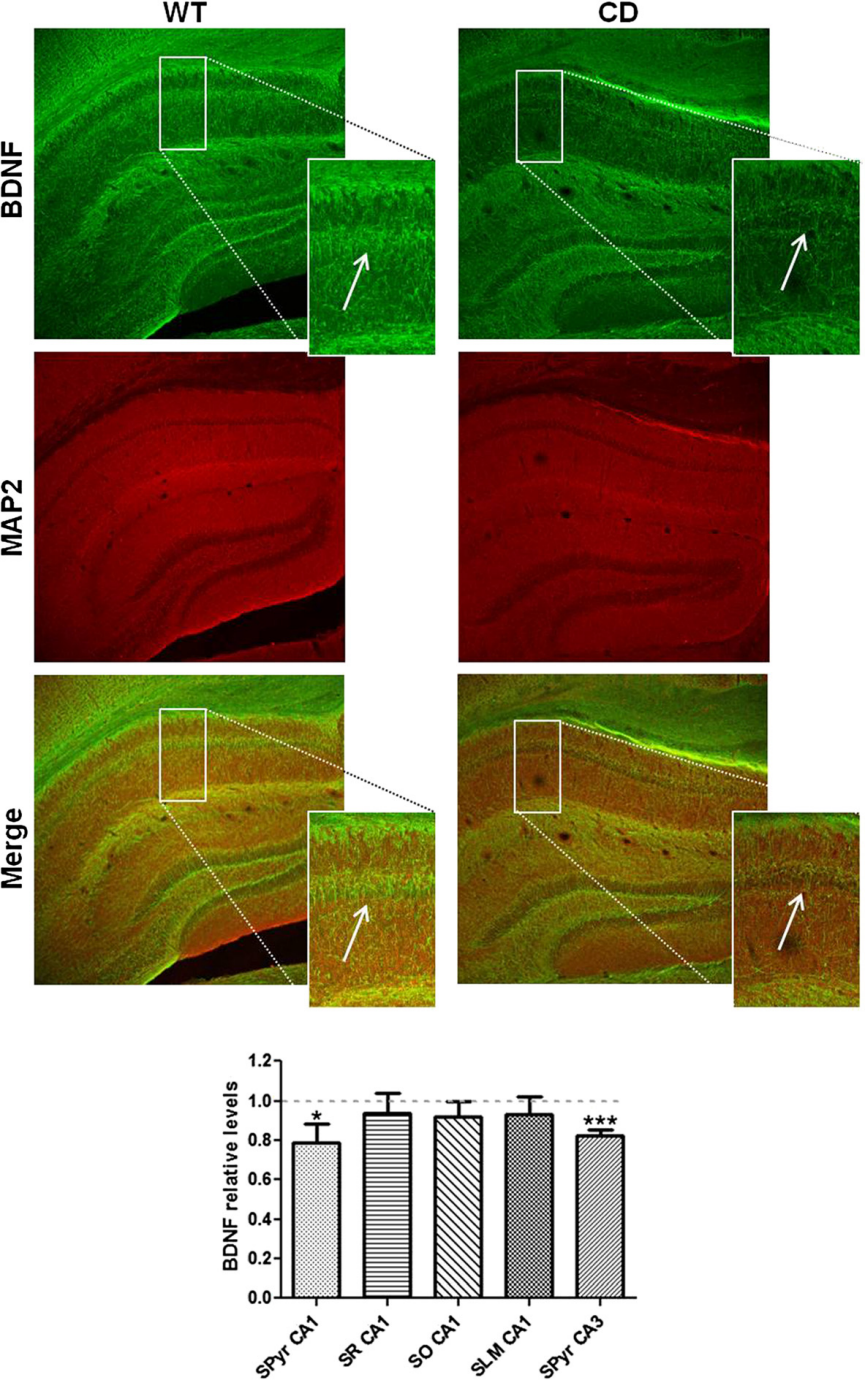
CD mice present decreased levels of BDNF in the hippocampal region. Representative immunofluorescence coronal sections through CA1 area of the hippocampus. BDNF fluorescence in pyramidal cells was measured with ImageJ software and then normalized to MAP2 intensity. 2-4 different regions per mouse were analyzed. BDNF levels in the soma of CA1 and CA3 neurons of CD mice were decreased compared to WT mice (*p* = 0.04 in CA1 and *p* = 0.0002 in CA3, unpaired *t* test). WT: *n* = 3, CD: *n* = 2. Data are presented as the mean ± SEM. *p* values are shown with asterisks indicating values that are significantly different. *p < 0.05; ***p < 0.001

Increasing evidence suggests that Brain-derived neurotrophic factor (BDNF) is a potent positive modulator of LTP and essential for the stabilization or consolidation of the potentiated state [36, 37]. Mutant mice deficient for BDNF displayed reduced LTP in the CA1 region of the hippocampus [27, 38]. In agreement with the reduced mRNA levels of *Bdnf* described in the hippocampus of CD animals [21], immunofluorescence assays showed reduced levels of BDNF, reaching significant differences in the soma of pyramidal neurons of CA1 and CA3 (*p* = 0.04 and *p* = 0.0002, respectively; unpaired *t* test) (Fig. 6). Therefore, this deficiency of BDNF could account for LTP deficits in CD mice.

## Discussion

LTP is considered the main cellular mechanism underlying learning and memory [39]. As such, this form of synaptic plasticity has been shown to be impaired in a wide variety of genetic models of neurological disease with cognitive deficits [40–43]. LTP deficits in the hippocampal CA1 region have been described in single gene knockout mice for genes included in the WBS region such as *Limk1*, *Clip2* and *Stx1a* [14, 16, 18, 19]. Here we also evaluated LTP at synapses between Schaffer collaterals and commissural neurons in CA1 of the hippocampus in the CD model of WBS. Our data show that CD mutant mice also present a significant reduction in LTP that correlates with memory impairment, consistent with the results obtained in the single genes knockout mice. Thus, the additive effects of haploinsufficiency at these genes might be in part responsible of the synaptic dysfunction observed in the CD model. In addition, these data highly suggest that individuals with WBS may also have deficits in hippocampal LTP as part of their complex phenotype.

In addition to synaptic plasticity alterations, all these single gene knockout models (*Limk1*, *Clip2* and *Stx1a*) exhibited similar memory deficits in the contextual fear conditioning test [14, 16, 18]. Similarly, CD mice showed impaired fear memory performance in the fear conditioning test, since they exhibited a slight reduction in the freezing time after the conditioning stimulus [11]. In this work, we showed that the LTP deficits observed in CD mice were also accompanied by cognitive dysfunction revealed by impaired spatial working memory. CD mice performed poorly in the spontaneous alternation test, a simple but demonstrated sensitive method in detecting hippocampal dysfunction [24], specifically deficits in spatial working memory [23, 25]. We chose a continuous trial procedure, in which the animal is left in the maze until it completes 15 trials/choices. In the discrete trial method, in which the mouse is placed back into the starting box after each choice, the constant handling between trials is stressful for the mouse and it can affect the results [44]. We found that CD mice displayed significantly reduced cognitive behavioral performance on the T-maze task compared to WT mice, without differences in locomotion levels. Therefore, the worse performance of CD mice in this test indicates spatial working memory impairment, which might be correlated with the synaptic plasticity defects observed in these mice.

Several LTP-related phenomena could be the responsible for the deficits observed in CD mice. A plausible explanation would be the presence of changes in the probability of transmitter release from the presynaptic terminal. Nevertheless, consistent with *Limk1* and *Stx1a* mutant mice [18, 45], the magnitude of PTP and the degree of PPF in CD mice were indistinguishable from WT. Changes in LTP have been related to alterations in mEPSCs frequency in *Limk1* knockout neurons [18]. However, in agreement with electrophysiological studies performed in human induced pluripotent stem cell derived neurons of a WBS individual [46], we could not appreciate any change in the frequency or amplitude of mEPSCs, suggesting that LTP deficits in CD mice cannot be attributed to presynaptic defects or changes in the biophysical properties of postsynaptic AMPA receptors. The study of theta bursts responses showed no differences between genotypes and our analysis of the AMPA/NMDA ratio suggests intact NMDA receptor function in CD mice. Collectively, these results point to a process other than initial induction as the element in LTP production that is affected in CD mice.

Given the above conclusion, it seems reasonable to hypothesize that CD mice exhibit defects in LTP maintenance and expression mechanisms that result in a reduced ability of CA1 synapses to sustain the increase in synaptic strength after the theta burst stimulation. There is overwhelming body of evidence demonstrating that BDNF plays an important role in synaptic plasticity [47–49]. Specifically, BDNF expression seems to have a crucial role in generating sustained structural and functional changes at hippocampal synapses by activating multiple pathways [50]. Mutant mice deficient for BDNF exhibited a significantly reduced LTP in the CA1 region of the hippocampus [27, 51]. In addition, several cognitive diseases have associated deficits in *Bdnf* expression or BDNF signaling with LTP and memory disturbances [40, 43, 52]. Significantly decreased mRNA levels of *Bdnf* have been documented in the hippocampus of CD mice [21]. We showed that protein levels of this neurotrophin are also reduced in the hippocampus of CD mice. Patterson and colleagues demonstrated that BDNF is required for the late LTP (L-LTP) produced by theta burst stimulation. BDNF appeared to modulate the translocation of the activated MAPK from the dendrites to the soma and then to the nucleus, where it has access to several nuclear substrates that contribute to the persistence of potentiation, such as the transcription factor cAMP response element binding (CREB) [53]. Curiously, the absence of LIMK1 resulted in reduced plasticity-dependent CREB activation, and by increasing the activity of CREB, LTP and memory deficits in *Limk1* knockout mice could be rescued [19]. All together these data indicate that deficiencies of BDNF and LIMK1 present in the CD model could account for the LTP deficits via plasticity-dependent CREB activation, and suggests that LTP deficits in WBS might be treatable by enhancing CREB activation and/or BDNF signaling in the brain. On the other hand, the PI3K pathway, which has been demonstrated to be deregulated in several cognitive diseases [54–56] is also activated by BDNF. Interestingly, WBS has also been related to the PI3K pathway since the regulatory subunit of PI3K (*Pik3r1*) is a direct target of GTF2I [57], previously described as a good candidate for the neurobehavioral features of WBS [12, 13].

In addition to molecular mechanisms, LTP is accompanied by changes in cytoskeletal organization and in the morphology of dendritic spines [58]. Dendritic spines contain the majority of excitatory synapses on CA1 pyramidal neurons and changes in spine density or morphology have been associated with aberrations in synaptic plasticity [18, 59, 60]. CD mice present a reduction in spine density in apical proximal dendrites of CA1 pyramidal neurons, fact that share with single knockout mice of *Gtf2i* [11, 21]. *Clip2* and *Limk1* are two synaptic genes that regulate cytoskeletal dynamics, either via the actin filament system (LIMK1) or through the microtubule network (CLIP2) [17]. Alterations in gene dosage of these molecules may lead to defects in neuronal structure and hence, in synaptic plasticity. In fact, *Limk1* knockout mice presented abnormal morphology of dendritic spines of pyramidal neurons, which correlated with alterations in LTP [18]. It is thus possible that spine abnormalities found in CD mice contribute to disrupt the production of LTP. Therefore, cytoskeletal defects might be involved in the neurological symptoms of WBS patients.

To sum up, this study highlights the utility of the CD model to study the mechanisms underlying the complex WBS neurocognitive profile. We report that LTP elicited by TBS was significantly impaired in hippocampal field CA1 of CD animals, which may contribute to the cognitive and behavioral phenotype of these mice. LTP deficit was not associated with changes in presynaptic function, LTP induction or AMPA and NMDA receptor function. Important issues for future investigation raised by this study will include determining the potential mechanistic underpinnings of synaptic plasticity deficits in CD mice, which might be related to BDNF-related–(MAPK and PI3K) pathways. It will be extremely important to further study the functioning of these pathways since they open new potential therapeutic approaches, currently unavailable for WBS.

## Methods

### Animals

Generation and initial characterization of CD mice were described previously [11]. Mice were maintained on at least 97 % C57BL/6 J background. During the experimental phase, mice were housed under a standard 12/12 light/dark cycle condition with access to food and water *ad libitum*. Genotype was determined using multiplex ligation-dependent probe amplification (MLPA) and appropriate primers [21]. All protocols and experimental procedures used for this study were approved by the local Committee of Ethical Animal Experimentation (CEEA-PRBB).

### Behavioral testing

Behavioral testing was performed in adult males of 2–3 months of age. All the experiments were performed during the light phase of the dark/light cycle by researchers unaware of the different experimental groups. The spontaneous alternation test was conducted in a T-maze. A central partition extending from the centre of the T into the start arm was included, allowing access to only one goal arm at a time and forcing the mouse to return to the starting arm each time [25]. The maze was equipped with one removal guillotine door separating a compartment at the beginning of the start arm. Mice were individually placed in the start compartment of the T-maze and after 5 s of confinement, the start arm door was lifted allowing mice to freely choose between the two goal arms (A or B). A total of 15 free choices made by the mice were annotated and the percentage of alternation during the free choice trials was calculated. The time to complete the 15 trials was also recorded and analyzed. After every single mouse, the apparatus was cleaned with a diluted ethanol solution.

### Electrophysiology

#### Whole cell recordings

Mice were sacrificed by cervical dislocation at P18-P25. The brain was quickly removed and immersed in ice-cold dissection buffer (in mM: 2 KCl, 0.5 CaCl2, 7 MgCl2, 1.3 NaH2PO4, 26 NaHCO3, 20 D-glucose, 200 sucrose, saturated with 95 % O2 and 5 % CO2) that was continuously oxygenated. Sagital hippocampal slices (330 μm) were cut with a vibrotome (Leica VT1200S). Slices containing the hippocampus were recovered incubating them for 30 min in warm (35 °C) and oxygenated artificial cerebral spinal fluid (aCSF) (in mM): 125 NaCl, 2.5 KCl, 2 CaCl_2_, 1 MgCl_2_, 1.2 NaH_2_PO_4_, 25.9 NaHCO_3_, 20 D-glucose, saturated with 95 % O_2_ and 5 % CO_2_ and with an osmolarity of 305-315 mOSm. Afterwards, they were maintained at room temperature until required. A single slice was then placed in a recording chamber with oxygenated aCSF at a rate of 1.5 mL/min. Whole cell recordings were performed on CA1 pyramidal hippocampal neurons identified with a differential interference contrast microscope (Eclipse FN-1, Nikon) equipped with an infrared camera (VX 44, Till Photonics) using an Axopatch-200B amplifier (Axon Instruments). Signals were filtered at 2 kHz and digitized at 5 kHz via a DigiData 1322A interface (Axon instruments).

Patch clamp electrodes (3–5 MΩ) were pulled out from borosilicate glass (GF 150 F-10) and were filled with a cesium methanesulfonate-based solution (in mM): 133 CsMeSO_3_, 2 MgCl_2_, 4 NaCl, 3 ATPNa_2_, 0.4 GTP, 5 phosphocreatine, 10 HEPES and 0.2 EGTA. A giga seal was achieved by gentle suction and the membrane was ruptured by further brief suction. Recordings were discarded if the resistance changed by >20 % over the course of the experiment. A minimum of 10 cells from a minimum of 4 animals were recorded per genotype.

For PPF, two pulses of equal intensity with an interstimulus interval of 50 ms were applied at Schaffer collateral afferents in the presence of bicuculline (10 μM) added to aCSF to inhibit GABA-A receptors. The membrane potential of CA1 neurons was clamped at −70 mV and fifteen consecutive responses were recorded. The ratio was obtained by dividing the amplitude of the second excitatory postsynaptic currents (EPSC) by the amplitude of the first EPSC.

To record mEPSCs, tetrodotoxin (0.5 μM) and bicuculline (10 μM) were added to the bathing solution to block action potentials and GABAergic transmission through GABA-A receptors, respectively. Slices were recorded at 30 °C. The membrane potential was clamped at −70 mV and mEPSCs were recorded for twenty minutes. Data were analyzed with Neuromatic in conjunction with Igor Pro software using a threshold of 7 pA and all detected events were manually checked. Amplitude and frequency of the events for each cell were measured.

For the AMPA/NMDA ratio, Schaffer collateral afferents were stimulated at 0.1 Hz in the presence of bicuculline (10 μM). AMPA receptor-mediated current was recorded at −70 mV. Holding potential was then changed to +40 mV to record NMDA receptor currents. Twenty consecutive responses were recorded on each condition and were averaged. The AMPA receptor current amplitude was calculated at the peak at a holding potential of −70 mV and the NMDA receptor current amplitude was measured 40 ms after stimulation artifact at a holding potential of +40 mV, a time point at which AMPA receptor-mediated currents are absent. The ratio was obtained by dividing the AMPA by the NMDA receptor current amplitude. To compare decay times of NMDA, we used a weighted mean decay time constant: tw 5 [If /(If 1 Is)] * tf 1 [Is/(If 1 Is)] * ts.

#### Field recordings

Mice aged 6 to 8 weeks were anesthetized with isoflurane and decapitated according to institutional regulations. Brains were removed to a chilled sucrose based solution (in mM: 215 sucrose, 2.5 KCl, 26 NaHCO_3_, 1.6 NaH_2_PO_4_, 1 CaCl_2_, 4 MgCl_2_, 4 MgSO_4_, 20 glucose) and coronal brain slices (350 μm thick) were cut with a Vibratome Series 3000 Plus-Tissue Sectioning System (Ted Pella, Inc). Sections containing the hippocampus were allowed to recover by incubating them for 30 min at 32 °C in a solution containing the following (in mM): 62 NaCl, 2.5 KCl, 25 NaHCO_3_, 1.4 NaH_2_PO_4_, 1.1 CaCl_2_, 3.3 MgCl_2_, 2 MgSO_4_, 15 glucose and 108 sucrose. Slices were then stored for at least 1 h at 32 °C in aCSF (in mM): 124 NaCl, 2.5 KCl, 25 NaHCO_3_, 1.2 NaH_2_PO_4_, 2.5 CaCl_2_, 1.3 MgCl_2_ and 10 glucose). Experiments were conducted in a submersion-type recording chamber perfused at 1.5 mL/min with aCSF at 32 °C. All solutions were saturated with 95 % O_2_ and 5 % CO_2_, pH 7.4.

fEPSPs were recorded from CA1 stratum radiatum using a borosilicate pipette filled with aCSF (1–2 MΩ). Synaptic responses were elicited by stimulation of the Schaffer collateral afferents with 150 μs duration pulses delivered through a bipolar platinum-iridium stimulation electrode (FHC, CE2C55). Input-output curves were constructed by plotting fEPSP slopes as a function of stimulation intensities ranging from 0 to 140 μA in increments of 20 μA. PPF was assessed using a succession of paired-pulses using inter-pulse intervals of 50, 100, 150 and 200 ms and calculated as the ratio of the second field potential slope to the first field potential slope.

After the PPF study, baseline stimulation was delivered at ~40 % of the maximum fEPSP for at least 20 min to ensure stability of the response. LTP was induced by using a TBS which comprised 5 trains of 10 theta bursts -each containing 4 pulses at 100 Hz with an interburst interval of 200 ms- delivered 15 s apart. Responses were recorded for 1 h after plasticity induction and fEPSP slopes were calculated for analysis and expressed as a function of averaged baseline responses. The size of composite postsynaptic responses induced by TBS was determined by analyzing the bursts area. To evaluate theta train facilitation, responses to the second burst in each train were expressed as a percentage change from the area of the initial burst response.

Group size values presented in the figures represent number of slices tested. Generally, two to three slices were tested from a given mouse, and no fewer than three mice were used in any group. Recordings were performed with a MultiClamp 700B (Axon Instruments), and output signals were filtered at 1 KHz. Data were digitized (10 KHz) on a DigiData 1332A (Axon Instruments) and collected using Clampex 9.2 and fEPSP initial slopes and area were analyzed using Clampfit 9.2.

### Immunohistofluorescence and imaging

Adult mice were transcardially perfused with PBS and then with 4 % paraformaldehyde in PBS. The brains were removed and postfixed for 24 h at 4 °C with 4 % paraformaldehyde in PBS. Next, they were cryoprotected in 30 % sucrose, frozen on dry ice, and sectioned on a cryostat (40 μm thick). Coronal sections were permeabilized for 10 min at room temperature with 1 % Triton X-100 in PBS. After washing in PBS, sections were blocked for 1 h at room temperature in a blocking solution (1 % Triton X-100 in PBS and 2 % bovine serum albumin-fraction V). Primary antibodies [polyclonal antibody BDNF (1:100, Santa Cruz Biotechnology) and monoclonal antibody MAP2 (1:500, Sigma-Aldrich)] were diluted in the same blocking buffer and slices were incubated overnight at 4 °C. After washing in PBS, secondary antibodies [Alexa Fluor 555 goat anti-mouse IgG (H + L) and Alexa Fluor 488 goat anti-rabbit IgG (H + L) (Invitrogen) at a concentration of 1:1000)] were diluted in 2 % bovine serum albumin (fraction V) and 3 % fetal bovine serum and slices were incubated for 1 h at room temperature in the dark. Slices were finally mounted on a glass slide in a drop of Mowiol after washing in PBS. For fluorescence quantification, the mean gray value of the region of interest was measured with ImageJ. BDNF fluorescence in CA1 and CA3 pyramidal cells was measured and then normalized to MAP2 intensity.

### Statistical analyses

All data are presented as means ± SEM. Statistical significance was assessed using unpaired *t* test and two-way repeated measures ANOVA. Kolmogrov-Smirnov test was used for cumulative distribution of inter-event intervals and amplitude of mEPSCs. GraphPad Prism software was used for all statistical tests and graphs.

## Abbreviations

aCSF, artificial cerebral spinal fluid; BDNF, brain-derived neurotrophic factor; CD, complete deletion; CREB, cAMP response element binding; fEPSP, field excitatory postsynaptic potential; IPSP, inhibitory postsynaptic potential; IQ, intelligence quotient; LTP, long-term potentiation; mEPSCs, miniature excitatory postsynaptic currents; MLPA, multiplex ligation-dependent probe amplification; PPF, paired-pulse facilitation; PTP, post-tetanic potentiation; TBS, theta burst stimulation; WBS, Wiliams-Beuren syndrome; WT, wild-type

## Additional file

Additional file 1: Figure S1. PPF of EPSCs was unchanged in CD mice. (DOCX 54 kb)
