# Supplementary material for: Synaptic plasticity and spatial working memory are impaired in the CD mouse model of Williams-Beuren syndrome
Source: Mol Brain. 2016 Aug 2;9:76. doi: 10.1186/s13041-016-0258-7 (PMC4971717; doi:10.1186/s13041-016-0258-7)
Supplement: Additional file 1: Figure S1. — PPF of EPSCs was unchanged in CD mice. (DOCX 54 kb) [file 13041_2016_258_MOESM1_ESM.docx]

**Additional file**

**
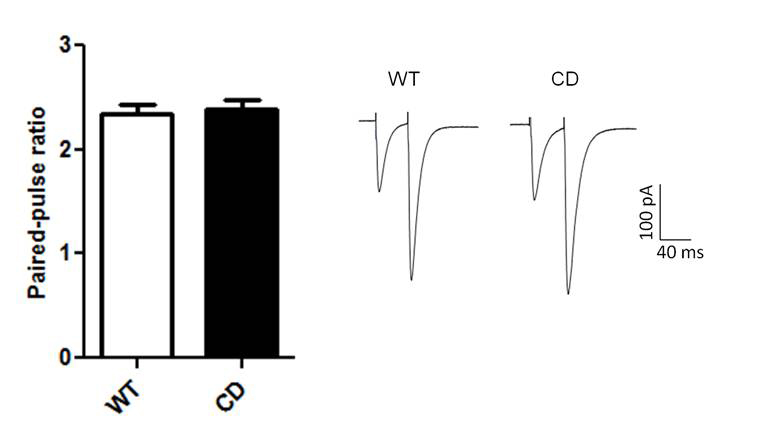
**

**Figure S1** PPF of EPSCs was unchanged in CD mice. Facilitation of excitatory currents was examined using an interpulse interval of 50 ms. Comparison of PPF in between WT and CD mice did not reveal differences between genotypes (WT: n=18, CD: n=14; *p*=0.770, unpaired *t* test). Representative traces of PPF are shown. Data are presented as the mean ± SEM.
